# Patterns of Peripartum Depression and Anxiety During the Pre-Vaccine COVID-19 Pandemic

**DOI:** 10.21203/rs.3.rs-2294673/v1

**Published:** 2022-12-13

**Authors:** Marie R Altendahl, Liwen Xu, Ifeyinwa Asiodu, John Boscardin, Stephanie L Gaw, Valerie J Flaherman, Vanessa L Jacoby, Misty C Richards, Deborah Krakow, Yalda Afshar

**Affiliations:** University of California, Los Angeles; University of California, Los Angeles; University of California, San Francisco; University of California, San Francisco; University of California, San Francisco; University of California, San Francisco; University of California, San Francisco; University of California, Los Angeles; University of California, Los Angeles; University of California, Los Angeles

**Keywords:** pregnancy, COVID-19, depression, anxiety, maternal mental health

## Abstract

**Background:**

Pregnant people are vulnerable to new or worsening mental health conditions. This study aims to describe prevalence and course of symptomatic depression and anxiety in pregnancy during the pre-vaccine COVID-19 pandemic.

**Methods:**

This is a prospective cohort study of pregnant individuals with known or suspected COVID-19. Participants completed Edinburgh Postnatal Depression Scale (EPDS) and Generalized-Anxiety Disorder-7 (GAD-7) questionnaires at 34weeks gestational age, 6–8weeks postpartum, and 6months postpartum. Prevalence of symptomatic depression and anxiety at each visit was described. Univariable logistic regression analysis was used to determine the association between demographic and clinical factors and symptomatic depression or anxiety

**Results:**

317 participantswere included. The prevalence of antepartum depression was 14.6%, 10.3%, and 20.6% at 34weeks gestational age, 6–8weeks postpartum, and 6months postpartum, respectively. The rate of anxiety was 15.1%, 10.0%, and 17.3% at 34weeks gestational age, 6–8weeks postpartum, and 6months postpartum, respectively. A prior history of depression and/or anxiety (p’s<0.03), as well as higher EPDS and GAD-7 scores at enrollment (p’s<0.04) associated with depression and anxiety throughout pregnancy and the postpartum period. Quarantining during pregnancy was associated with symptomatic anxiety at 34weeks gestational age in univariate (P=0.027) analyses. COVID-19 diagnosis and hospitalization were not associated with depression or anxiety.

**Conclusions:**

Depression and anxiety were prevalent throughout pregnancy and the postpartum period, particularly in those with prior depression and/or anxiety and who quarantined. Strategies that target social isolation may mitigate potential adverse consequences for pregnant people, and continued vigilance in recognition of depression and anxiety in pregnancy should be considered.

## Introduction

The COVID-19 pandemic has provoked significant fear, uncertainty, and stigma in the perinatal population. Implementation of measures intended to curb the spread of the virus such as social and physical distancing, travel bans, and lockdowns have engendered unprecedented isolation^1^. The prevalence of anxiety and depressive symptoms were found to be increased during previous infectious disease outbreaks^2,3^, including among pregnant people^4^. During the peripartum period women are especially vulnerable to experiencing the onset or relapse of psychiatric disorders^5^. Whereas the prevalence of depression and anxiety is 3–5% in the general population^6^, an estimated 13–23% of women suffer from depression or anxiety during pregnancy and postpartum^7,8^, up to 9% incidence of comorbid depressive and anxiety symptoms^9^. Untreated antenatal depression and anxiety have both been associated with adverse perinatal outcomes such as preterm delivery and low birth weight^10,11^, as well as impaired mother-infant bonding and delayed childhood cognitive/emotional development^12–14^.

To date, data has been unclear on whether, in the context of the COVID-19 pandemic, pregnant and postpartum individuals experience more depression and anxiety symptoms^15–19^. Hessami et al. showed that perinatal anxiety scores were higher during the COVID-19 pandemic among peripartum individuals and that peripartum individuals had higher pooled validated depression scores during the pandemic compared to pre-pandemic, but the difference was not significant^20^. A more recent meta-analysis by Shorey et al. suggested a higher prevalence of depressive symptoms in the antepartum period during COVID-19, but no difference in the prevalence of depressive symptoms in the postpartum period^21^. One small case series of 14 pregnant women with laboratory-confirmed SARS-Cov-2 infection reported similar levels of depression and anxiety compared to 14 matched non-infected pregnant women^22^. Pregnant people may be in even higher need of preventive mental health interventions during the COVID-19 pandemic. We describe the prevalence of depressive and anxiety symptoms among pregnant people with known or suspected COVID-19 throughout the peripartum period.

## Methods

PRIORITY (Pregnancy CoRonavIrus Outcomes RegIsTrY) is a prospective cohort study of pregnant or recently pregnant people with known or suspected COVID-19 infection. Participants were recruited nationwide between March 2020 and October 2020 through outreach by professional societies, community organizations, traditional media, and social media. Eligible participants were ≥ 13 years old, spoke any language, pregnant or within 6weeks of pregnancy, and under investigation for COVID-19 infection or had confirmed COVID-19 diagnosis. Participants were provider-referred or self-referred. Informed consent was obtained from each individual participant in accordance with study protocols approved by the University of California, San Francisco (UCSF) and the University of California, Los Angeles (UCLA) Institutional Review Boards (IRB) (UCSF IRB# 20-30410, UCLA IRB# 20-000579). PRIORITY follow up is ongoing; for this manuscript, we report data available through August 18, 2021.

Baseline demographic and clinical characteristics were collected at the time of enrollment. Demographic characteristics included race/ethnicity, region of residence (Midwest, Northeast, South, West), language, sexual orientation, relationship status, employment status, and annual income. Clinical characteristics included age, BMI, substance use history, medical history, gestational age, obstetric history, antepartum/intrapartum complications, neonatal outcomes, COVID-19 diagnosis, and COVID-19-related hospitalization. Self-report of SARS-CoV-2 diagnosis was adjudicated with viral test results on a subsample of 140 participants; 138 (98.6%) were concordant with self-report.

Participants completed questionnaires about their health, pregnancy history, COVID-19 diagnosis, and quarantine practices at enrollment, weekly for 4weeks, and then at multiple time points throughout pregnancy and postpartum. These questionnaires also included Edinburgh Postnatal Depression Scale (EPDS) and Generalized Anxiety Disorder-7 (GAD-7). For this analysis, we selected PRIORITY participants who enrolled while pregnant < 24 weeks gestation and completed the EPDS and GAD-7 at least once antepartum (24 weeks and/or 34 weeks gestational age) and once postpartum (6–8weeks postpartum and/or 6months postpartum). Symptomatic depression was assessed using the EPDS. EPDS scores of ≥ 13 yielded a sensitivity of 88% and specificity of 93% postpartum for probable cases of major and minor depression^24^, and the same cutoff has also been validated for use during pregnancy^25^. Symptomatic anxiety was assessed using the GAD-7 scale. GAD-7 score of ≥ 10 yielded a sensitivity of 89% and specificity of 82% for generalized anxiety disorder independently diagnosed by mental health professionals^26^, with good reliability and construct validity in pregnancy and the postpartum period^27^. Severity of depression or anxiety was measured using the quantitative score of EPDS or GAD-7, respectively. Those with higher scores on EPDS or GAD-7 were interpreted as having more severe depression or anxiety.

Statistical analysis was performed using SAS version 9·4, R version 3·6·2, and Stata 15. Descriptive statistics were used to summarize baseline demographic and clinical characteristics for the total study population and COVID-19 positive subgroup. The prevalence of symptomatic depression and anxiety was reported 24weeks gestation, 34weeks’ gestation, 6–8weeks postpartum, and 6 months postpartum. Severity of depression and anxiety throughout pregnancy and postpartum was approximated using the mean EPDS scores and GAD-7 scores. Repeated measures linear and logistic regression models were used to examine change in prevalence and severity of depression and anxiety over time; time point was treated as a categorical fixed effect and random intercepts were included for each subject to account for the correlation of the repeated measures. Univariable logistic regression analysis was used to determine the association between demographic and clinical factors and symptomatic depression or anxiety at 34 weeks’ gestation, 6–8weeks postpartum, and 6 months postpartum. Maternal demographic variables of age, parity, region of residence, relationship status, employment status, and annual income were considered in a multivariable logistic regression model. We calculated summary statistics and 95% confidence intervals (CI). Statistical significance was set at P-value of 0·05.

## Results

**Figure 1:**
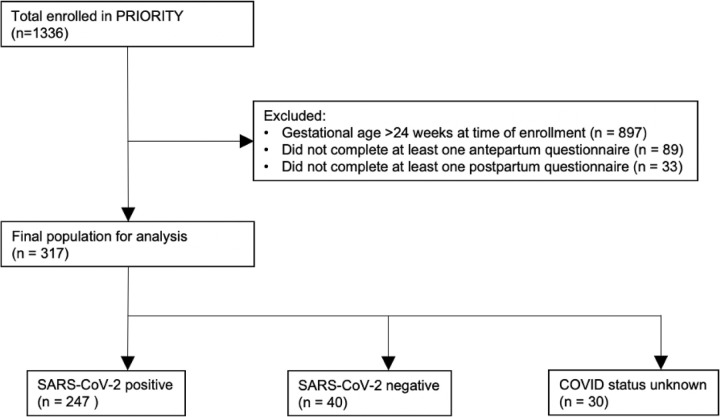
Diagram representing the selection of final study population and associated COVID-19 diagnoses.

Of 1,336 PRIORITY participants, 317 enrolled prior to 24weeks gestation and completed questionnaires both in the antepartum and postpartum period and thus were included in the study (Fig. 1). Among the 317 individuals with completed questionnaires, 247 tested positive for SARS-CoV-2, and 40 were negative.

Mean maternal age of the sample was 31.5 (standard deviation [SD] 4.91) with 41.0% being nulliparous (Table 1). Mean weeks of gestation was 17.1 (SD 5.43) at enrollment and 38.6 (SD 2.82) at birth. Nearly all pregnancies were singleton (99.0%) and resulted in livebirths (98.7%). Two individuals (1.5%) reported having considered an abortion. Most of the cohort (62.8%) identified as White and 27.8% identified as Hispanic/Latinx, living in the Midwest (17.3%), the Northeast (28.1%), the South (25.8%), and the West (30.7%). Notably, 47.0% reported an annual income of more than $100,000. In this cohort, 26.5% reported a history of depression, anxiety, or both. Other pre-pregnancy medical comorbidities were reported by 30.9% of the cohort. Only 2.2% of the participants were hospitalized at time of enrollment, but more than half (60.9%) self-reported they were in quarantine at time of enrollment. Demographic and clinical characteristics for participants who tested positive for SARS-CoV-2 infection are also presented in Table 1.

**Figure 2:**
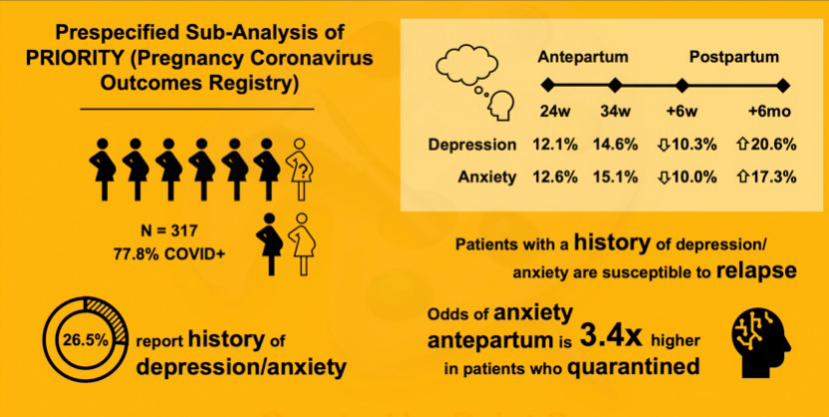
Summary of key study results: COVID-19 positivity rate among study participants, prevalence of a prior history of depression and anxiety, prevalence of symptomatic depression and anxiety throughout the perinatal period, and the relationship between anxiety and quarantine practices.

In this peripartum cohort, rate of symptomatic depression and anxiety as defined by validated cutoffs of EPDS and GAD-7 scores was lowest at 6–8weeks postpartum and highest at 6months postpartum (Table 2). The prevalence of symptomatic antepartum depression was 12.1% (95% CI: 7.6–17.9%) at 24 weeks gestational age and 14.6% (95% CI: 10.8–19.1%) at 34 weeks. Rate of symptomatic depression was lower (10.3%, 95% CI: 7.2–14.3%) at 6–8weeks postpartum, and increased (20.6%, 95% CI: 15.7–26.2%) by 6 months postpartum (Fig. 2). For symptomatic antepartum anxiety, the prevalence was 12.6% (95% CI: 8.1–18.5%) at 24 weeks and 15.1% (95% CI: 11.2–19.7%) at 34weeks. Similarly, rate of symptomatic anxiety was lower (10.0%, 95% CI: 6.9–13.9%) at 6–8weeks postpartum and rose again to 17.3% (95% CI: 12.7–22.6%) by 6months postpartum (Fig. 2). At 24weeks, 16.1% (95% CI: 11.0–22.4%) of the cohort met criteria for both symptomatic depression and symptomatic anxiety. 20.6% of participants had comorbid (past medical history of) depression and/or anxiety (95% CI: 16.2–25.6%) at 34weeks, 13.5% (95% CI: 9.9–17.9%) at 6–8weeks postpartum, and 26.3% (95% CI: 20.9–32.3%) at 6 months postpartum.

The results of univariable logistic regression analyses examining the association of demographic and clinical factors with peripartum depression are presented in Supplemental Table 1A. A prior history of depression and/or anxiety, as well as higher EPDS and GAD-7 scores at enrollment were significantly associated with symptomatic depression at 34weeks gestation, 6–8weeks postpartum, and 6months postpartum. At 6–8 weeks postpartum, increasing age was protective for symptomatic depression (OR 0.92, 95% CI 0.85–0.99, P = 0.021). In the multiple logistic regression analyses (Supplemental Table 2A), prior history of depression and/or anxiety was associated with increased odds of depression at 34weeks’ gestation (OR 6.8, 95% CI 2.9–15.7, P < .0001), 6–8 weeks postpartum (OR 8.5, 95% CI 3.1–23.1, P < .0001), and 6months postpartum (OR 2.4, 95% CI 1.1–5.2, P = 0.03). Higher GAD-7 score at enrollment was associated with increased odds of depression at 34weeks (OR 1.4, 95% CI 1.14–1.7, P = 0.001) and at 6–8 weeks postpartum (OR 1.2, 95% CI 1.0–1.4, P = 0.026), but not at 6 months postpartum. Higher EPDS score at enrollment was significantly associated with 1.6-fold increased odds of depression only at 34 weeks’ gestation (OR 1.6, 95% CI 1.2–2.1, P = 0.002). At 34weeks’ gestation, hypertensive disease of pregnancy (OR 3.2, 95% CI 1.1–8.9, P = 0.028) and “Other” antepartum complications (OR 3.5, 95% CI 1.1–11.0, P = 0.034) were both associated with approximately three-fold increased odds of depression.

Similar univariate logistic regression analyses were conducted for peripartum anxiety. The findings are summarized in Supplemental Table 1B. Symptomatic anxiety at 34 weeks’ gestation and postpartum time points was associated with a prior history of depression and/or anxiety and higher enrollment EPDS and GAD-7 scores. Antepartum complications (P = 0.003) predicted anxiety at 34 weeks. Hispanic/Latinx identity was significantly associated with anxiety at 6–8 weeks postpartum (OR 2.6, 95% CI 1.2–5.9, P = 0.019). Both employment status (P = 0.034) and annual income (P = 0.043) were associated with increased odds of anxiety at 6 months postpartum. For the multivariate analyses (Supplemental Table 2B), prior history of depression and/or anxiety was associated with increased odds of anxiety at 34weeks’ gestation (OR 4.0, 95% CI 1.9–8.6, P = 0.0003), 6–8 weeks postpartum (OR 10.4, 95% CI 3.5–31.0, P < .0001), and 6 months postpartum (OR 3.7, 95% CI 1.6–8.8, P = 0.002). Higher GAD-7 at enrollment was also associated with increased odds of anxiety at all time points (at 34 weeks’ gestation: OR 1.8, 95% CI 1.3–2.3, P < .0001; at 6–8weeks postpartum: OR 1.3, 95% CI 1.0–1.6, P = 0.018; and at 6months postpartum: OR 1.5, 95% CI 1.1–1.9, P = 0.005). Higher EPDS score at enrollment was significantly associated with increased odds of anxiety at 34 weeks’ gestation (OR 1.5, 95% CI 1.2–1.9, P < .0001) and 6 months postpartum (OR 1.3, 95% CI 1.0–1.6, P = 0.024), but not at 6–8weeks postpartum. Additionally at 34 weeks’ gestation, antepartum complications of gestational diabetes (OR 4.0, 95% CI 1.3–12.7, P = 0.019), hypertensive disease of pregnancy (OR 3.2, 95% CI 1.1–9.0, P = 0.028) and “Other” antepartum complications (OR 3.7, 95% CI 1.3–10.6, P = 0.014) were associated with approximately 3–4 fold increased odds of anxiety.

Pandemic-specific factors such as COVID positive status, hospitalization, observation of quarantine did not predict symptomatic depression in the univariate (Supplemental Table 1A) or multivariate (Supplemental Table 2A) analyses. Interestingly, quarantine for COVID at present was associated with significantly increased odds of anxiety at 34weeks gestation in both the univariate analysis (OR 2.5, 95% CI 1.1–5.6, P = 0.027) and the multivariate analysis (OR 2.7, 95% CI 1.0–7.1, P = 0.040). At 6–8 weeks postpartum, the association between anxiety and quarantine practices were not significant in the univariate analysis. However, multivariate analyses adjusting for maternal demographic variables, anxiety was predicted by both quarantine at enrollment (OR 3.1, 95% CI 1.1–8.7, P = 0.04) and quarantine at present (OR 3.5, 95% CI 1.1–10.1 P = 0.03) at 6–8weeks postpartum.

## Discussion

### Clinical Implications

Symptomatic depression and anxiety were prevalent throughout pregnancy and postpartum during the COVID-19 pandemic, irrespective of patient SARS-CoV-2 status. Participants who had a history of depression and/or anxiety, as well as those with higher baseline EPDS and GAD-7 scores, were more susceptible to symptomatic depression and anxiety during pregnancy and postpartum. Participants who quarantined during pregnancy had higher odds of symptomatic anxiety antepartum but not postpartum. On the other hand, those who quarantined during pregnancy did not have increased prevalence of depression. Interestingly, unlike findings in the general population, our participants who tested positive for COVID-19 during pregnancy or were hospitalized during their pregnancy were not more likely to report symptomatic depression and anxiety, though our analyses may be underpowered^28^. We describe the prevalence of depression and anxiety among pregnant patients throughout the peripartum period, including those with confirmed SARS-CoV-2 infections, and identify risk factors associated with peripartum depression and anxiety during the COVID-19 pandemic.

In our study, the prevalence of symptomatic depression and anxiety across the peripartum period ranged between 10% and 20%. This is comparable to pre-pandemic rates of peripartum depression and anxiety based on meta-analyses ^7,8^; but lower than published rates of peripartum depression and anxiety during the COVID-19 pandemic^29^. The difference may be partially attributable to differences in EPDS cut-offs for clinical significance. Wu et al. reported 29.6% prevalence of peripartum depression as defined by EPDS score ≥ 10, but subgroup analysis showed 13.9% had EPDS score ≥ 13, which was aligned with our findings. Nevertheless, in a meta-analysis of eight studies by Hessami et al., the overall mean EPDS score during the pandemic was 9.84, and in our cohort the mean EPDS score was between 5.79 at 6–8 weeks postpartum and 7.45 at 6 months postpartum, lower than those reported in the meta-analysis ^18,20^. With regards to anxiety, pooled prevalence for perinatal anxiety across four studies was 50% in a meta-analysis by Shorey et al., noting high heterogeneity between studies ^21^. Our cohort may have lower reported rates of symptomatic depression and anxiety compared to Hessami et al. and Shorey et al. because we looked prospectively at the prevalence of depression or anxiety at specific antepartum and postpartum timepoints, not just at any point during the peripartum period. Our cohort includes participants enrolled between March and October 2020, while Hessami et al. looked at participants in February 2020. It is possible that participants enrolled later in the pandemic may have lower rates of depression and anxiety, than those at the beginning of the pandemic. In the general population, mental health conditions such as depression and anxiety did decrease as the pandemic progressed ^30–33^. Since our cohort included participants enrolled at later time points compared to Hessami et al. it is expected that we would have lower rates of reported depression and anxiety.

Our study found that the prevalence of both symptomatic depression and anxiety increased with gestational age during pregnancy, decreased at 6–8weeks postpartum, and peaked by 6months postpartum. In a meta-analysis published pre-pandemic, depression was more prevalent as pregnancy continued, finding that the average prevalence of depression in the first trimester of pregnancy was 7.4% and increased to 12.0–12.8% by the second or third trimester ^34^.

### Research Implications

Given the impact of depression and anxiety on maternal and neonatal health outcomes, it is imperative for clinicians to identify which pregnant patients are at highest risk for developing depression and anxiety during the COVID-19 pandemic. Our study found that participants at the highest risk for depression and anxiety during the COVID-19 pandemic included those with a prior history of depression and/or anxiety, observing quarantine, or of Hispanic/Latinx identity. In our cohort, those with prior depression and/or anxiety or those with higher baseline EPDS and GAD-7 scores were more likely to have symptomatic depression and anxiety at all antepartum and postpartum timepoints. Prior depression and/or anxiety are well-known risk factors for major depressive disorder with peripartum onset, and the COVID-19 pandemic may contribute further to peripartum depression and anxiety. Moyer et al. found that pregnant individuals with a history of depression or anxiety pre-pandemic were most likely to experience the largest increases in anxiety during the COVID-19 pandemic ^35^. This finding is also seen in the general population. Numerous studies have shown that in the general population, those with pre-existing mental health conditions similarly reported higher rates of depression or anxiety during the COVID-19 pandemic ^32, 36–38^ Furthermore, our participants who quarantined during pregnancy had higher rates of anxiety, but not depression, in the antepartum period. Pregnant patients who felt feelings of isolation had increased rates of symptomatic depression and anxiety and those with better perceived social support actually had lower rates of depression and anxiety ^39^. Sommerland et al. found that in 71,117 UK-dwelling participants, those with daily face-to-face or phone/video contact were less likely to report depressive symptoms compared to participants with little social contact, yet this is a non-pregnant population, unlike our study^36^. Our study findings emphasize that in pregnant people with known or suspected COVID-19 pre-existing mental health conditions and/or social isolation are important risk factors for development symptomatic depression and anxiety throughout the peripartum course during the pre-vaccine COVID-19 pandemic. The impact on telehealth during COVID-19 should be explored and how this could modify maternal health and/or attenuate risk factors. Thus, it is imperative for health care providers to ask about mental health history in the obstetrics space to identify those at greatest risk for peripartum depression and anxiety.

In our cohort, Hispanic or Latina identity was significantly associated with increased likelihood of symptomatic anxiety at 6–8 weeks postpartum. Birthing persons of color have higher rates of postpartum mental health conditions, but are often under screened and under counseled, compared to White persons ^40,41^. Specifically, in a study by Declercq et al, only 18.3% of Latina participants with prenatal depressive symptoms were counseled prenatally on postpartum depression compared to 43.4% of White participants ^41^. With racial/ethnic inequities related to mental health screening and access to mental health services among birthing people, our study emphasizes the necessity to screen and counsel all participants for anxiety in antepartum and postpartum period.

### Strength and Limitations

Our study does have limitations. We defined symptomatic depression and anxiety using EPDS and GAD-7 cut-off scores, not provider-validated clinical diagnoses. EPDS and GAD-7 are validated screening tools frequently used to screen for depression and anxiety in both the research and clinical settings. Past validation studies estimate the sensitivity of EPDS ranging between 65–100% and specificity between 49–100% ^42^ and estimate the GAD-7 as having a sensitivity of 89% and specificity of 82% ^43^. Although EPDS and GAD-7 are validated screening tools for depression or anxiety, especially for research purposes, they are not diagnostic and thus we may not have accurately identified all participants with clinically significant anxiety/depression in our study. EPDS and GAD-7 do not directly investigate pregnancy-related depression and anxiety. Pregnancy-specific anxiety is a better predictor of poorer birth outcomes compared to general anxiety^21^. Understanding how concerns surrounding childbirth, infant health, and parenting concerns during the COVID-19 pandemic and how they contribute to anxiety and depression experienced during the peripartum period need further exploration, particularly if they impact maternal/neonatal birthing outcomes. We also had challenges establishing a baseline mental health assessment, as participants did not complete the EPDS and GAD-7 pre-pandemic. As such were unable to investigate precisely how the COVID-19 pandemic, including length of hospitalization and visitor policies directly impacted our participants’ mental health. Our study was also limited by the fact that we did not consider how treatment with antidepressants or anxiolytics may change participants EPDS or GAD-7 scores. Participants treated with antidepressants and anxiolytics possibly have lower EPDS and GAD-7 and as such, might not have been categorized into “symptomatic” depression or anxiety subgroups. Going forward, we hope to gather this information for future studies.

## Conclusions

Depressive and anxiety symptoms were prevalent throughout pregnancy and the postpartum period, particularly in those with prior depression and/or anxiety. Individuals who quarantined during pregnancy were more likely to experience antepartum anxiety. Strategies that target social isolation may mitigate potential adverse consequences for pregnant people, and continued vigilance in recognition of depression and anxiety in pregnancy should be considered.

## Supplementary Material

Supplement 1

## Figures and Tables

**Table 1:** Demographic and clinical characteristics of study participants

Characteristics	All participants	COVID + Subgroup
Age (years; mean ± SD (n))	31.5 ± 4.91 (n = 316)	31.3 ± 4.83 (n = 246)
Gestational age at enrollment (weeks; mean ± SD)	17.1 ± 5.43 (n = 317)	17.2 ± 5.44 (n = 247)
*Race/Ethnicity*		
Asian	22 (6.9%)	13 (5.4%)
Black	23 (7.3%)	19 (7.7%)
Hispanic/Latinx	88 (27.8%)	77 (31.2%)
White	199 (62.8%)	149 (60.3%)
Other	8 (12.8%)	7 (2.8%)
*Language*		
English	297 (94.3%)	228 (93.1%)
Spanish	17 (5.4%)	16 (6.5%)
Other	1 (0.3%)	1 (0.4%)
*Region of country*		
Midwest	53 (17.3%)	43 (17.9%)
Northeast	80 (26.1%)	63 (26.3%)
South	79 (25.8%)	67 (27.9%)
West	94 (30.7%)	67 (27.9%)
*Employment status*		
Full time paid employment	223 (70.3%)	173 (70.0%)
Homemaker, childcare provider, or both (unpaid)	26 (8.2%)	21 (8.5%)
Part time employment or seeking employment	39 (12.3%)	30 (12.1%)
Healthcare worker or direct patient care	99 (31.2%)	83 (33.6%)
*Annual income ($)*		
Less than 25,000	24 (7.6%)	18 (7.03%)
25,000–50,000	46 (14.5%)	40 (16.2%)
50,000–100,000	88 (27.8%)	69 (27.9%)
More than 100,000	149 (47.0%)	112 (45.3%)
In a significant relationship (including marriage or domestic partner)	299 (94.3%)	236 (95.6%)
Heterosexual or straight	305 (96.2%)	237 (96.0%)
*Medical history*		
No pre-existing conditions	85 (26.8%)	71 (28.7%)
Medical co-morbidities	98 (30.9%)	74 (30.0%)
Mental health co-morbidities	84 (26.5%)	60 (24.3%)
Tobacco, alcohol, and/or marijuana use	16 (5.0%)	7 (2.8%)
*Body mass index*		
Less than 25	142 (45.5%)	102 (42.1%)
25–30	93 (29.8%)	69 (28.5%)
30 or higher	77 (24.7%)	71 (29.3%)
Nulliparity	130 (41.0%)	101 (40.9%)
IVF pregnancy	32 (10.1%)	27 (10.9%)
*Antepartum complications*		
Gestational diabetes	23 (7.4%)	19 (7.9%)
Hypertensive disease of pregnancy	38 (12.3%)	32 (13.3%)
Oligo/polyhydramnios or PPROM	26 (8.4%)	18 (7.5%)
Other	34 (11.0%)	24 (10.0%)
Intrapartum complications	71 (22.6%)	51 (20.8%)
Gestational age at delivery (weeks; mean ± SD)	38.6 ± 2.82 (n = 314)	38.5 ± 3.09 (n = 245)
*Pregnancy outcome*		
Live birth of an infant	310 (98.7%)	241 (98.4%)
Abortion	2 (0.6%)	2 (0.8%)
Miscarriage	1 (0.3%)	1 (0.4%)
Death of an infant > 20 weeks	1 (0.3%)	1 (0.4%)
NICU admission	40 (12.6%)	29 (11.7%)
Infant “roomed in” after delivery	268 (84.5%)	208 (84.2%)
Hospitalized at enrollment	7 (2.2%)	7 (2.8%)
Quarantined at enrollment	193 (60.9%)	165 (66.8%)

**Table 2:** Prevalence of peripartum depressive and anxiety symptoms across the antepartum and postpartum periods. GA = gestational age.

	24 week GA	34 week GA	6–8 weeks postpartum	6 months postpartum	P-value
EPDS score: mean, SD (n)					
All participants	7.06 ± 4.73 (174)	7.24 ± 4.89 (301)	5.79 ± 5.01 (310)	7.45 ± 5.11 (243)	< .0001
COVID + subgroup	6.82 ± 4.92 (130)	6.95 ± 4.84 (235)	5.55 ± 4.96 (241)	7.29 ± 5.16 (189)	< .0001
*EPDS score: median (range)*					
All participants	7 (0.0–20)	7 (0.0–21)	5 (0.0–25)	6 (0.0–25)	--
COVID + subgroup	6.5 (0.0–20)	6 (0.0–20)	5 (0.0–25)	7 (0.0–20)	--
*Symptomatic Depression: n (%)*					
All participants	21 (12.1%)	44 (14.6%)	32 (10.3%)	50 (20.6%)	0.0026
COVID + subgroup	13 (10.0%)	32 (13.6%)	20 (8.3%)	37 (19.6%)	0.0009
*GAD-7 score: mean, SD (n)*					
All participants	4.63 ± 4.03 (174)	5.07 ± 4.49 (298)	3.93 ± 4.44 (309)	5.16 ± 5 (243)	< .0001
COVID + subgroup	4.59 ± 4.2 (130)	4.97 ± 4.63 (233)	3.66 ± 4.33 (240)	5.06 ± 5.07 (189)	< .0001
*GAD-7 score: median (range)*					
All participants	4 (0.0–19)	4 (0.0–21)	3 (0.0–21)	4 (0.0–21)	--
COVID + subgroup	4 (0.0–19)	4 (0.0–21)	2 (0.0–21)	4 (0.0–21)	--
*GAD score >/= 10: n (%)*					
All participants	22 (12.6%)	45 (15.1%)	31 (10.0%)	42 (17.3%)	0.014
COVID + subgroup	17 (13.1%)	36 (15.5%)	22 (9.2%)	33 (17.5%)	0.0056
*Comorbid depression/anxiety: n (%)*					
All participants	28 (16.1%)	62 (20.6%)	42 (13.5%)	64 (26.3%)	< .0001
COVID + subgroup	19 (14.6%)	45 (19.1%)	29 (12.0%)	49 (25.9%)	< .0001
