# Supplementary material for: Patterns of Peripartum Depression and Anxiety During the Pre-Vaccine COVID-19 Pandemic
Source: Res Sq. 2022 Dec 13:rs.3.rs-2294673. Preprint. [Version 1] doi: 10.21203/rs.3.rs-2294673/v1 (PMC9774217; doi:10.21203/rs.3.rs-2294673/v1)
Supplement: Supplement 1 [file NIHPPRS2294673v1-supplement-1.pdf]

Supplementary Files

This is a list of supplementary files associated with this preprint. Click to download.

- SupplementalTable1a.docx
- SupplementalTable1b.docx
- SupplementalTable2A.docx
- SupplementalTable2B.docx
